# A Case of Castleman Disease: A Diagnostic Dilemma

**DOI:** 10.7759/cureus.14372

**Published:** 2021-04-08

**Authors:** Ghulam Rabbani Anwar, Muhammad Tariq Mehr, Danial Tahir, Sidra Humayun, Ghulam Farooq

**Affiliations:** 1 Internal Medicine, Hayatabad Medical Complex Peshawar, Peshawar, PAK; 2 Internal Medicine, Ayub Medical College, Abbottabad, PAK; 3 Pathology, Muhammad College of Medicine, Peshawar, PAK

**Keywords:** castleman disease, extrapulmonary tuberculosis, systemic lupus erythematosus, lymphadenopathy, imcd, tuberculosis

## Abstract

Castleman disease (CD) is a disorder characterized by lymphoid proliferation. It is not usually the first differential for pyrexia of unknown origin (PUO) because of the extremely rare incidence worldwide. We report the case of a 24-year-old man with PUO for six months. He had been previously investigated for infective, rheumatological, and immunological causes. Extrapulmonary tuberculosis was considered as the most likely diagnosis because of his clinical presentation and locality. Based on this, he was given a trial of anti-tuberculous therapy. However, he did not show any signs of improvement despite being compliant with the medications. His condition was further complicated by the development of ascites. Upon treatment failure, the patient presented to our tertiary care hospital and was investigated for a possible revision of diagnosis. Based on clinical assessment and histopathology of the lymph nodes, he was diagnosed with idiopathic multicentric CD overlapping with systemic lupus erythematosus. He was started on azathioprine and prednisone and showed a positive response, indicated by a decreasing erythrocyte sedimentation rate and C-reactive protein. The patient continues to be healthy and in remission to date.

## Introduction

In clinical practice, disease manifestations are quite complex with a tremendous overlap. Tuberculosis (TB) is one such disease that can have a myriad of presentations. Extrapulmonary tuberculosis (EPTB) can have central nervous system and pleural manifestations as well as gastrointestinal and lymph node involvement. It is one of the top differentials in a patient with pyrexia of unknown origin (PUO) along with gastrointestinal and nodal involvement in an endemic region like Pakistan [1,2]. On the other hand, Castleman disease (CD) is a rare condition [3], characterized by non-clonal lymphoid proliferation. It has three histological types (i.e., hyaline vascular, plasma cell, and mixed) and two clinical subtypes (i.e., unicentric and multicentric) [4]. Systemic lupus erythematosus (SLE) is a chronic autoimmune disease affecting multiple organ systems including the lymph nodes. The most common lymph node lesions in SLE are follicular hyperplasia and coagulative necrosis, with the latter being more specific for the disease [5]. Here, we present a case of a 24-year-old male with idiopathic multicentric Castleman disease (iMCD) overlapping with SLE.

## Case presentation

A 24-year-old male patient from the northwestern region of Pakistan presented to the outpatient clinic of our tertiary care hospital with an unexplained fever of six months. He had been using anti-tuberculous therapy (ATT) for four months for EPTB based on the previous workup for PUO. Although he had been compliant with his medications, his symptoms did not improve. Therefore, he came to our hospital for a possible revision of the diagnosis. He had four to five episodes daily of high-grade fever, characterized by sweating, rigors, and chills. The fever was not restricted to a specific time of the day and was partially alleviated with antipyretics. It was associated with a headache that was mild in intensity, throbbing, involving the full head, and affecting daily activities. The patient also gradually developed abdominal distension over the previous 40 days. On presentation to our hospital, the patient appeared pale, depressed, and in obvious discomfort. Significant abdominal distension was also noted. Physical examination revealed bilateral flank fullness, dull percussion notes, shifting dullness, and a positive fluid thrill. After the initial assessment, he was started on diuretics and all baseline investigations were sent. After confirming ascites on ultrasound, the ascitic fluid was sent for analysis, serum ascites albumin gradient (SAAG) ratio and culture, for a possible local etiology. About 3 L of ascitic fluid were removed via paracentesis for symptomatic relief. His lab reports revealed him to be anemic with a hemoglobin level of 5.9 g/dL (normal range for men = 13.5-17.5g/dL), mean cell volume of 75.1 fL (normal range = 80-96 fL), and a normal white blood cell count of 8,690/uL (normal range = 4,500-11,000/uL). His platelet count was low at 73,000/uL (normal range = 150,000-450,000/uL), a high erythrocyte sedimentation rate (ESR) of 60 mm/first hour (normal range = 0-15 mm/first hour), and a raised C-reactive protein (CRP) of 34 mg/L (normal range = less than 10 mg/L). Testing for human immunodeficiency virus, hepatitis B virus, hepatitis C virus, cytomegalovirus, and Epstein-Barr virus was negative. Repeated blood and urine cultures were requested which grew no microorganisms.

**Figure 1:**
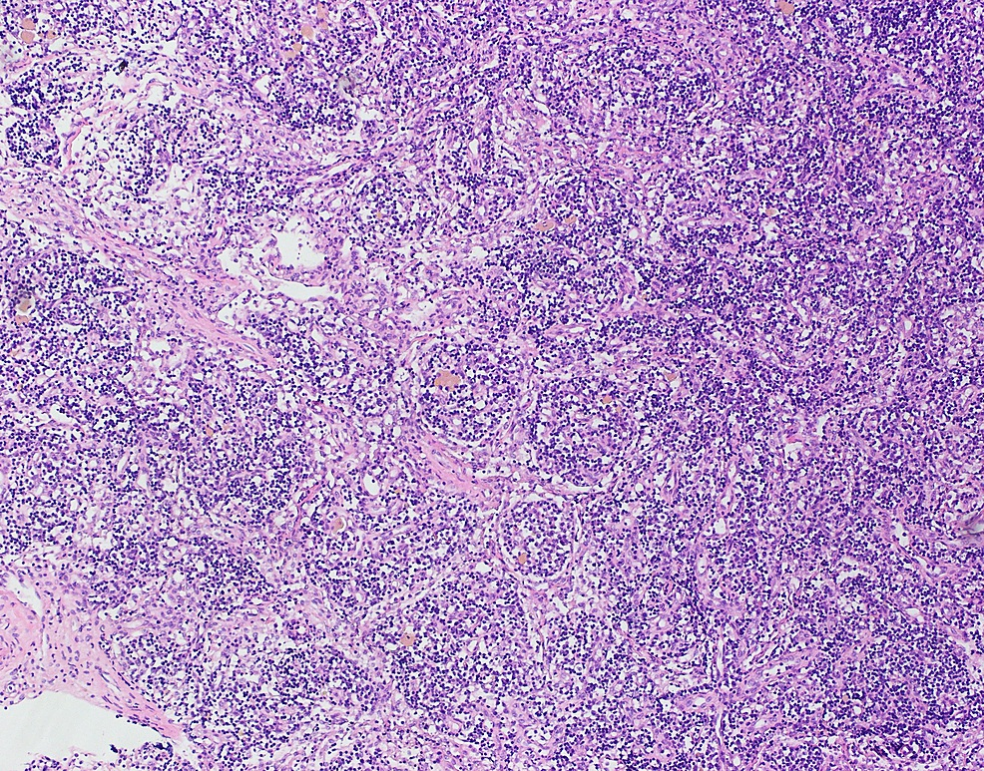
Vascular proliferation and hyalinization of vessels and lollipop follicles.

**Figure 2:**
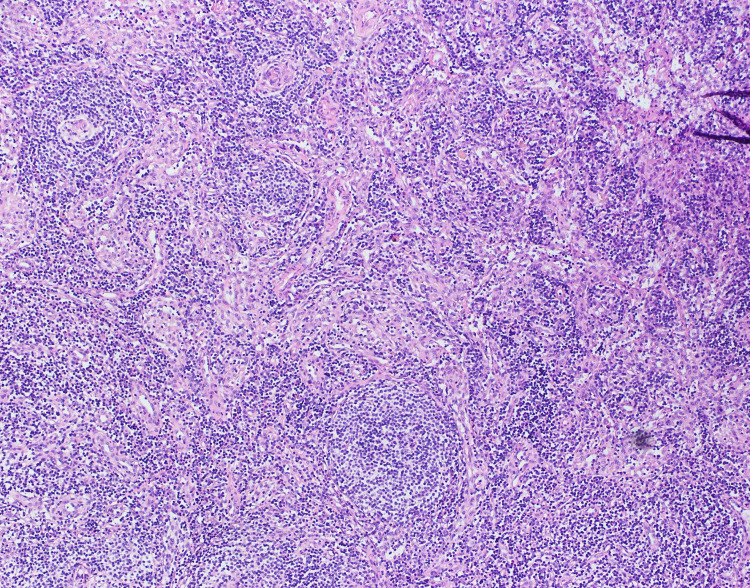
Onion skin appearance: mantle zone thickened with lymphocytes arranged in layers.

The ascitic fluid analysis showed 4.9 g/dL of protein (normal range = less than 3 g/dL) and 200 cells/uL (normal range = less than 500 cells/uL), 85% of which were lymphocytes and neutrophils making up the remaining 15%. No microorganisms were detected in the fluid, including acid-fast bacilli. Ascitic fluid had a low SAAG ratio of 0.8 mg/dL with an adenosine deaminase level of 6.5 U/L (normal range = 8.6-20.5 U/L). Ascitic fluid culture grew no organisms and GeneXpert for *Mycobacterium tuberculosis* was also negative. Markers for autoimmune diseases were requested; antinuclear antibodies (ANA) were positive (>1:80) in the nucleolar pattern with low levels of C3 and C4, suggesting an autoimmune disease like SLE. Surprisingly, anti-dsDNA and anti-Smith antibodies along with the rest of the extractable nuclear antigen (ENA) panel came back negative. A previous report of a contrast-enhanced computed tomography (CT) scan of the abdomen and pelvis revealed para-aortic and inguinal lymphadenopathy. After much consideration, a laparoscopy was done for lymph node biopsy. Histopathology report was consistent with CD, the hyaline vascular type. Findings included prominent vascular proliferation and hyalinization of vessels and germinal zones traversed by penetrating vessels, lollipop follicles (Figure 1), and mantle zone thickened with lymphocytes arranged in layers, onion skin appearance (Figure 2). Furthermore, HHV-8 testing was done which was negative.

Our patient’s histopathology report was indicative of iMCD and had a 10-point score on the European League Against Rheumatism (EULAR)/American College of Rheumatology (ACR) criteria for SLE. His clinical findings could be explained by both MCD and SLE. Therefore, a diagnosis of iMCD overlapping with SLE was made. The patient was started on appropriate therapy with prednisone and azathioprine. He was asked to come back after two weeks for a follow-up. At the two-week follow-up, his condition had started showing signs of improvement, as indicated by the clinical outlook, regressing ascites, and normalizing inflammatory markers (CRP: 17 mg/L, ESR: 8 mm/first hour). The corticosteroid dose was tapered and the azathioprine dose was increased to the target of 2.5 mg/kg per day. He was advised to come for a follow-up again in three months. At the next follow-up, his condition was markedly improved, he had put on weight and rejoined his academic activities. We are regularly following the patient and he has been doing well thus far.

## Discussion

TB is one of the main etiologies of PUO in endemic regions. It can be a challenging disease to diagnose, particularly when it involves extrapulmonary sites [6]. Therefore, every effort should be made in this regard to avoid misdiagnosis or inadequate and/or suboptimal treatment. Moreover, other causes of PUO such as autoimmune diseases and malignancies with shared clinical features should be thoroughly investigated [7]. EPTB can affect any organ of the body, with the lymph nodes and gastrointestinal tract being common sites [6]. Our patient was initially diagnosed with EPTB based on the length of fever and lymphadenopathy and was empirically given a trial of ATT [7]. However, he did not show signs of improvement and presented to us for a revision of the diagnosis. A stepwise approach to the investigation was adopted which helped us reach a final diagnosis.

SLE is a chronic autoimmune disease affecting multiple organs of the body. Autoimmune diseases affect the male population at a much lower rate compared to the female population [8]. SLE has a similar prevalence with every ninth or tenth patient being a male [9]. Our patient scored 10 points on the 2019 EULAR/ACR criteria for SLE, along with positive ANA with fever, thrombocytopenia, and low C3 and C4 levels [10]. The most common staining pattern of ANA in SLE is homogenous, while nucleolar is the rarest pattern [11]. ENA panel of our patient showed ANA in nucleolar pattern, as well as negative anti-dsDNA and anti-Smith antibodies.

CD is a rare condition characterized by non-clonal lymphoid proliferation. It has three histological types, namely, hyaline vascular, plasma cell, and mixed. Based on the number of lymph node regions involved, there are two clinical subtypes. If one nodal region is involved, it is called unicentric Castleman disease (UCD) [4]. UCD is the most common variant [3]. CD is classified as MCD when two or more regions of lymph nodes are involved. MCD can be secondary to HHV-8 (HHV-8 associated MCD) or idiopathic (iMCD) [4]. The diagnosis of iMCD requires exclusion of infection with HHV-8 and autoimmune diseases along with other conditions outlined in the exclusion criteria [12]. However, the presence of autoantibodies without a definitive autoimmune diagnosis should not exclude iMCD because autoantibodies including ANA were found in ∼30% of iMCD patients in the largest series reported to date [12]. In our patient, the negative workup for virology paired with typical features on lymph node biopsy was indicative of a diagnosis of CD. CD was further classified as iMCD based on the number of nodal regions involved and a negative HHV-8 result. Our patient’s clinical course was rare due to several reasons: the simultaneous occurrence of CD with SLE, a young male having SLE, and the presence of only ANA with negative anti-dsDNA and anti-Smith antibodies.

The clinical and pathologic manifestations of iMCD are diverse and tend to mimic multiple other conditions, signifying that numerous processes may give rise to it [13]. Whether it is possible that a condition like SLE could precede the disease or exist simultaneously with iMCD needs to be investigated further.

## Conclusions

Although TB is highly prevalent in the Indo-Pak subcontinent, it is not always the culprit. There are other conditions with similar presentations that one needs to keep among the differentials. The updated diagnostic criteria for iMCD necessitates excluding autoimmune disorders such as SLE before making a final diagnosis. Sometimes, however, an overlap of these two conditions cannot be avoided confidently. We are hopeful that future studies will address this overlapping issue and bring forth more clarity.
